# Supplementary material for: Are carbon emissions trading and green financial instruments synergistic? -Comprehensive quantitative research based on content analysis
Source: PLoS One. 2024 Mar 7;19(3):e0298601. doi: 10.1371/journal.pone.0298601 (PMC10919601; doi:10.1371/journal.pone.0298601)
Supplement: S1 Data — (ZIP) [file pone.0298601.s001.zip › Data/地级市Z_F工作报告.zip!/╜¡╦╒/╠⌐╓▌╩╨/└┤╫╘╣1⁄2╓┌║┼í╛┬φ┐╦╩2╛▌═°í┐_2014.docx]

各位代表：

　　现在，我代表市人民政府向大会作工作报告，请予审议，并请市政协委员和其他列席人员提出意见。

一、 2013年工作回顾

　　过去的一年，我们在中共泰州市委的正确领导下，深入学习贯彻党的十八大、十八届三中全会和习近平总书记系列重要讲话精神，全面落实“三项要求”、“八项工程”、“十项举措”，以提高经济发展质量和效益为中心，实施“双轮驱动”战略，加快“三个名城”建设，推进转型升级融合发展，经济社会发展稳中有进，较好完成全年各项目标任务。

　　经济实力进一步增强。预计实现地区生产总值3020亿元，增长12%；公共财政预算收入259.3亿元，增长11.2%；固定资产投资1760亿元，增长21%；居民消费价格涨幅1.9%。农业现代化步伐加快。国家现代农业示范区建设有力推进，市区北部现代农业走廊规划完成编制和评审，姜堰成为全省唯一的国家农业改革与建设试点地区，泰兴农业产业园标准化示范区通过国家验收。新增高效设施农业19万亩、高效设施渔业2.6万亩、高标准农田18.1万亩。工业经济稳中向好。规模以上工业增加值2000亿元，增长13%；实现主营业务收入8100亿元、利税1000亿元、利润600亿元，分别增长19%、18%、18%。完成工业投资960亿元，增长21%。新增销售10亿元以上企业27家、利税过亿元企业11家；新增私营企业10272家、个体工商户26448户，净增私营个体经济注册资本636.3亿元。建筑业总产值2159亿元，工程结算收入1450亿元，分别增长15.6%、18.5%。服务业发展较快。实现增加值1245亿元，增长12.2%；社会消费品零售总额833亿元，增长13%。八大省级现代服务业集聚区获省重新评定。凤城河风景区成为全省唯一的国家级城市中央休闲区，溱湖风景区入选首批国家生态旅游示范区，兴化被评为全国休闲农业与乡村旅游示范县。全市接待游客1620万人次，实现旅游总收入197亿元。年末全市本外币存款余额3612.6亿元、贷款余额2464.2亿元，分别增长17.6%、18.4%，累计实现直接债务融资额120亿元，华夏、民生、浙商银行泰州分行开业。

　　转型升级成效凸显。以发展壮大“1+3+N”产业体系为重点，努力在加快结构调整中提质增效。优质项目支撑引领作用不断增强。组织实施转型升级“双百”工程，完成投资367.8亿元，65个项目竣工投产。扬子江药业注射剂、阿斯利康供应基地、可胜科技铝镁合金制品、伽力森食品加工等一批重大项目正在加快建设。新兴产业持续发力。深入实施“新兴产业双倍增计划”，我市被列为全国首批战略性新兴产业区域集聚发展试点，全年实现产值3306亿元，增长23.2%。生物技术与新医药、电子信息、新能源三大新兴产业实现利税200亿元、利润120亿元，分别增长32%、33%。高新技术产业产值3350亿元，增长23.7%，增幅高出规模以上工业5.3个百分点。传统产业逆势奋进。认真实施“传统优势产业转型升级五年规划”，装备制造业实现产值3260亿元，增长14.7%。船舶产业显现触底回升迹象，造船完工量、新接订单量均居全省首位。科技创新成效突出。研发经费支出占地区生产总值比重达2.15%。连续5次入选全国科技进步先进市，获批国家创新型试点城市、国家知识产权示范城市，新公示国家高新技术企业65家。加快科技成果转化，组建东南大学国家技术转移（泰州）中心，成立全省首家外籍院士工作站，新建产学研联合体147家、省级工程技术研究中心14家。加强知识产权保护，新获专利授权8000件，其中发明专利240件。积极打造人才高地，新增国家“千人计划”专家12名、省“双创计划”人才22名。强力推进节能减排，单位地区生产总值能耗下降4.5%，主要污染物排放量削减完成年度目标任务。

　　改革开放不断深化。转型升级综合改革试点扎实推进。与15家省级机关部门签订合作协议，各项工作全面展开、初见成效。深化行政审批制度改革，推进并联审批、联审联验，取消、调整三分之一审批事项。开展行政事业性收费专项清理，收费标准和项目进一步规范。公共资源交易中心基本建成。实施工商登记制度改革，推行个体工商户选择登记等新模式。创新投融资机制，实施企业授信总额联合管理，农信社全部改制为农商行。扩大环境污染责任保险投保范围，推进企业环保信用评价。启动事业单位分类改革，经济发达镇行政管理体制改革取得阶段性成效。开放型经济难中求进。全年新批外资项目155个，实际利用外资12.5亿美元。完成外贸进出口总额103亿美元，有进出口实绩企业比上年增加47家。实现外经营业额8.3亿美元，泰兴河海科技在印尼成功签约2个过亿美元项目。海关、国检、海事、边检为开放开发作出积极贡献。特色园区承载功能有效提升。中国医药城进入产业化发展转折期，新落户企业125家，在建产业化项目180个；泰州北京儿童医院签约落户，省体外诊断试剂检测中心、省食品药品检验所分所挂牌成立，获批全省首家药品专用公共型保税仓库，成功举办第四届医博会。出口加工区二期加快建设，纬立资讯启动实施，纬创进入产能释放期。新能源产业园林海电动车一期、智航锂离子材料正式投产，永邦重工钢结构、汇景导电玻璃等即将建成。沿江开发深入推进，靖江港口岸开放通过国家验收，核心港区永安港务5万吨级集装箱码头基本竣工。里下河特色经济板块后劲增强，兴化循环经济产业园、姜堰南洋电缆等重大项目进展顺利。

　　城乡发展加快融合。深入实施“城乡转型2115计划”和“美丽城乡建设行动”，积极推动城乡发展一体化。城市功能形象进一步提升。启动第四轮城市总体规划修编，做好姜堰撤市设区后相关规划调整，推进市区主干道联网贯通，“一城四区”格局初步形成。“九路一桥”改造基本竣工，金融服务区及人民医院、中医院新院加快建设。生活垃圾焚烧发电厂竣工试运营，完成6个老小区燃气改造。三水湾二期主体工程完工，福音、开泰等一批游园建成开放。调整优化市区公交线路设置，开通首批专用车道和泰姜专线。推进城市精细化管理，强化违法建设防控，市容环境持续改善。城乡基础设施建设不断加强。宁启铁路复线电气化改造进入收尾阶段，京沪高速江广段扩容、阜兴泰高速泰州段前期工作取得明显进展，229、233、334等省道建设顺利。卤汀河拓浚完工，泰东河整治、引江河二期等完成序时任务。实施农村新一轮实事工程，新改建道路367公里、桥梁204座，疏浚县乡村河道1589条。节水型社会建设试点通过水利部验收，农村饮水安全工程新增受益人口19.5万人。生态环境建设力度加大。姜堰通过国家级生态区技术评估，海陵创成省级生态区，高港通过省级生态区考核验收，靖江、泰兴通过省级生态市技术评估，新增19个国家级生态乡镇。城市环境综合整治启动实施，农村环境整治共完成3805个村庄整治任务。实施大气环境监测和重污染天气预警，狠抓重点流域水污染防治和饮用水源地专项整治。全市新增造林面积9.4万亩，市区新增绿地面积100万平方米。

　　社会建设得到加强。认真办好民生实事项目，全市财政用于民生方面的支出占公共财政预算支出比重达75.2%。人民生活继续改善。预计城镇居民人均可支配收入、农民人均纯收入分别达29230元、13870元，分别增长10%、11%。深化国家级创业型城市建设，加强重点人群就业帮扶，新增创业人员6.1万人、城镇就业9.9万人，城镇登记失业率2.16%。落实企业退休职工基本养老金、新农合筹资和城乡低保标准自然增长机制，启动城镇居民大病再保险，取消医疗救助起付线和病种限制。实施困难群众临时生活和自然灾害救助，扩大孤儿保障对象范围，发展慈善福利事业。推行老年人意外伤害综合保险，建成省级示范性社区居家养老服务中心21家。全市新建各类保障性住房12937套，住房公积金新增开户缴存职工4.6万人。推进新一轮扶贫开发，扶持发展村级集体经济，新增集体经营性收入30万元以上村160个。社会事业协调并进。获批国家历史文化名城，新增全国重点文物保护单位4家，黄桥老街入选中国历史文化名街。市文化馆、工人文化宫、青少年活动中心等重点文化设施建成开放，市档案馆创成国家一级综合档案馆。深化文明城市创建，推进道德讲堂建设，弘扬社会新风正气。大力发展教育事业，各级各类教育水平不断提高，靖江、兴化通过义务教育发展基本均衡县国家级评估验收，泰州师范高等专科学校升格为泰州学院，江苏农牧科技职业学院获评全国示范性高职院校优秀等级。整合市区医疗卫生资源，推进县级公立医院改革试点，强化基层医疗卫生机构建设，城乡公共卫生服务差距逐步缩小。加强人口计生工作，保障妇女儿童权益。市体育局荣获全国群众体育先进单位。社会科学、广播电视、民族宗教、外事侨务和气象、防震、无线电管理等工作扎实推进。社会保持和谐稳定。加大平安创建力度，获评全省平安市。实施社会稳定风险评估，开展领导干部接访下访和信访积案化解，预防和减少社会矛盾。强化基层基础工作，社会管理三级服务平台基本建成。推进“六五”普法，完善法律援助体系。加强基层民主法治建设，开展和谐社区创建，完成村居两委换届。应急管理体系不断健全，安全生产形势总体稳定。抓好双拥和征兵工作，退役士兵村官培养工程启动实施。

　　政务环境持续改善。深化拓展“三解三促”，组织开展“治四风、建四队”活动，深入基层解决实际问题。构建“三服务”长效机制，强化政策支持和要素保障，严格控制涉企检查，制定落实减负措施。推进绩效管理工作，完善指标体系和评估程序，初步形成“三分三统全覆盖”管理新模式。加快电子政务集约化建设，建成市数据政务中心，我市入选首批国家智慧城市试点。规范行政权力网上公开透明运行，政府信息公开和新闻发布工作有效开展。坚持依法行政，强化法治政府建设，自觉接受市人大及其常委会的法律监督、工作监督和市政协的民主监督，切实加强审计监督，广泛听取各民主党派、工商联、无党派人士和社会各界意见。做好人大代表建议和政协提案办理工作，落实率74%。整合开通“12345·政风行风热线”，认真办好市长信箱、望海楼实名论坛，深入开展纠风治乱，解决群众合理诉求。严格执行改进作风各项制度规定，落实党风廉政建设责任制，严肃查处违纪违法案件，公务员队伍建设和管理进一步加强。

　　过去一年的成绩来之不易，这是中共泰州市委正确领导的结果，是全市上下攻坚克难、团结奋斗的结果。我代表市人民政府，向全市广大干部群众，向全体人大代表、政协委员，向各民主党派、工商联、无党派人士、各人民团体和广大离退休老同志，向中央和省驻泰机构以及驻泰部队指战员、武警官兵和政法干警，向所有关心、支持泰州发展的海内外朋友，表示衷心的感谢！

　　我们也清醒地看到，经济社会发展中还存在一些问题和不足，主要是：受宏观形势影响，部分企业和行业运行困难增多，财政增收难度加大，城乡居民收入难以持续较快增长；土地、资金等要素制约日趋凸显，重大项目招引和实施难度加大；城乡统筹、区域统筹面临制约因素较多，区域发展不平衡不协调的问题仍然突出；绿色发展理念仍需强化，大气雾霾呈多发趋势，生态环境质量有待提升；政府自身建设还存在薄弱环节，机关作风和效能仍需进一步改进，等等。对此，我们一定高度重视，采取有效措施，切实加以解决。

二、 2014年工作任务

　　今年是转型升级“综合改革攻坚年”，也是全面完成“十二五”规划目标任务的关键年。政府工作的总体要求是：深入学习和全面贯彻党的十八大、十八届三中全会精神，按照中央和省经济工作会议、省委十二届六次全会和市委四届六次全会决策部署，牢固确立正确的政绩观、发展观，坚持稳中求进工作总基调，把改革创新贯穿于经济社会发展各个领域各个环节，更好更快推进转型升级融合发展，加快建设“三个名城”，努力实现经济持续健康发展、社会和谐稳定。

　　全市国民经济和社会发展主要预期目标为：地区生产总值增长10%左右；公共财政预算收入增长9%左右；固定资产投资增长18%左右；社会消费品零售总额增长13%左右；城乡居民收入增长与经济增长基本同步；城镇登记失业率控制在3.5%以内；居民消费价格涨幅3.5%左右；研发经费支出占地区生产总值比重达到2.3%；节能减排完成省定目标任务。

　　今年将重点抓好八个方面工作：

　　1. 加快转型升级，推进经济持续健康发展。围绕主题主线，按照“调高调轻调优调强”思路，努力构建具有泰州特色的现代产业体系，进一步提高经济发展的质量和效益。

　　加快中国医药城产业化步伐。紧扣“5个1000”目标，全力打造疫苗、诊断试剂及高端医疗器械、生物制药、化学药新型制剂、保健品、中药现代化等产业基地。实施德国勃林格殷格翰、瑞士雀巢、日本武田和中外制药、台湾桑缇亚、香港百草堂等一批重大产业化项目，推动阿斯利康、康淮生物、复旦张江等企业早日投产，全年新增量产医药企业100家以上。推进医疗器械集聚区、会展交易中心二期、数据中心三期等载体建设。积极申报国家药品进口口岸城市，加快省医药产业人才改革试验区建设。

　　大力发展先进制造业。以实施转型升级“双百”工程为抓手，新上一批高端产业项目和重点技改项目，淘汰一批落后产能项目，力争新兴产业产值达3900亿元。突出抓好“千企升级百企示范工程”，大力推进“两化融合”，力争年主营业务收入超50亿元企业达23家，新增上市企业2家。进一步优化中小企业发展环境，全面推进工商注册登记便利化，探索建立负面清单制度，放手发展民营经济。推进建筑业工业化，打响“建筑强市”品牌。

　　积极发展服务业。以现代物流、金融保险、软件和信息服务、服务外包等为重点，推动生产性服务业提速发展，力争占全部服务业比重提高1.5个百分点。实施“服务业集聚区提升计划”，新增百亿级集聚区1-2家。加快发展电子商务、信息消费、健康服务等新型业态。有序推进市区经济“退二进三”，发展总部经济、楼宇经济和服务型经济。完善商贸流通服务体系，优化商业网点布局，力促城乡商贸繁荣。

　　加强节能减排工作。强化源头控制，实施严格的环境准入标准，不再新上高能耗、高污染项目。认真落实监管措施，突出抓好建筑业、交通运输业等领域和船舶、化工等行业节能减排。推广应用节能减排新技术、新装备，发展绿色经济、循环经济。推进企业清洁生产和园区生态化改造，继续清理整治小化工、小电镀企业。大力促进资源节约高效利用，积极创建国家低碳试点城市。

　　2. 强化开放创新驱动，增创新的竞争优势。加快对内对外开放，促进技术创新和制度创新，不断激发经济发展的内生动力。

　　进一步提高对外开放水平。加大招商引资力度，重点引进技术含量高、污染少、能耗低、带动性强的重大项目。深入调整对外贸易结构，推动加工贸易向产业链高端延伸，大力发展服务贸易。扩大先进技术、关键设备和重要能源原材料进口。鼓励企业“走出去”，在境外开展资源开发、生产加工、品牌并购和建立营销网络。加强外事、侨务和港澳台事务工作，深化对外交流合作。

　　深入推进沿江开发开放。认真落实“沿江地区转型发展五年推进计划”，完善沿江港口规划和核心港区功能，统筹沿江产业带项目布局和岸线资源整合，发展优势临港产业。加快靖江三江现代物流园、高港综合物流园建设，打造沿江先进制造业和现代服务业“两业互动”示范区。加快沿江交通运输体系建设，开工建设泰镇高速、阜兴泰高速泰州段和京沪高速江广段扩容工程，完成宁启铁路复线电气化改造。主动融入苏南板块，发挥自身比较优势，实现与苏南的有效对接和错位发展。

　　加快特色园区建设。以20个特色园区为重点，进一步完善基础设施，提升产业承载、配套服务和环境保护功能。推进重点园区提档升级，加强与上海自贸区的对接融合，把泰州经济开发区作为自贸区的特殊功能区来定位、打造，支持出口加工区、靖江经济开发区争创综合保税区；支持泰兴、高港经济开发区争创国家级开发区；支持兴化经济开发区争创国家级食品加工区。推动有条件的园区与境内外高水平园区、知名企业开展多形式合作共建。

　　构建区域创新体系。抓住国家创新型试点城市建设机遇，推进“政产学研金”协同创新，进一步激发科技创新潜力。突出企业创新主体地位，实施“十百千创新型企业培育计划”，加快实现“两个高质量的全覆盖”。加强知识产权战略推进中心、江苏（泰州）医药技术产权交易中心等平台建设，培育各类专业孵化器，构建重点产业关键技术专利池，加快科技成果产业化。强化人才支撑，大力培养和集聚高层次创新创业人才、高水平管理人才、高技能实用人才。

　　3. 以经济体制改革为重点，深入推进转型升级综合改革试点工作。认真贯彻中央精神，按照省委、省政府和市委的部署，结合泰州实际，积极抓好重点领域和关键环节改革。

　　抓好经济体制改革。坚持统一管理、公开透明的原则，建成运行公共资源交易中心，制订和公布交易目录，规范交易程序和交易制度，真正使市场在公共资源交易和配置的过程中起决定性作用。以稳定和服务实体经济发展为重点推进金融改革，鼓励支持由民间资本发起设立中小型银行、金融租赁公司等金融机构，着力解决中小企业融资难、融资贵问题。以建立现代财政制度为方向推进财税体制改革，改进预算管理制度，稳步推进部门预算决算和“三公经费”公开，将政府性债务纳入全口径预算管理。加强政府融资平台管理，着力防范债务风险。深化国有企业改革，发展混合所有制经济，以管资本为主加强国有资产监管。

　　抓好行政管理体制改革。把行政审批制度改革作为重要突破口，对市场机制能有效调节、企业能自主决定、行业组织能自律管理的经济活动，逐步取消审批；对直接面向基层、由基层管理更方便有效的事项，有序下放基层。推行行政审批“三集中三到位”，进一步减少审批环节，提高审批效率。构建市区一体的扁平化管理机制，调整完善市区城建体制。推进重点园区和重点镇简政放权改革试点，赋予部分县级经济和社会管理权限。推进事业单位分类改革，调整理顺事业单位职能。

　　抓好社会领域改革。统筹推进教育领域综合改革，深化考试招生制度改革，申报建设国家中小学教育质量综合评价改革实验区。深入开展医药卫生体制改革，全面完成县级公立医院改革，加快推进乡镇卫生院和村卫生室一体化管理。调整食品药品监管体制，加大执法检查和质量监督力度，维护食品药品市场秩序。加快户籍制度改革，全面放开建制镇和小城市落户限制。

　　4. 保障和改善民生，着力维护社会和谐稳定。按照“守住底线、突出重点、完善制度、引导舆论”的要求，将财政资金、公共资源重点向民生领域和基层群众倾斜，让发展成果更多更公平惠及全体人民。

　　多渠道促进居民增收。加大农民创业就业、村集体经济发展、农民合作组织规范化建设推进力度，拓宽农民持续增收渠道。健全最低工资标准调整、工资正常增长和支付保障机制，扩大集体协商制度覆盖面，提高职工工资收入水平。以扩大就业夯实增收基础，强化就业服务平台建设和帮扶措施落实，促进重点群体充分就业，净增城镇就业4.4万人。以加强社会保障兜住低收入群体生活底线，建立与经济增长、物价水平相适应的临时救助和城乡低保标准调整机制，健全孤儿、残疾人等分类保障制度。统筹整合城乡居民基本养老、基本医疗保险等制度，有序推进五险合一征缴。强化住房保障和供应体系建设，增强公积金制度的住房保障作用。发展社会养老服务，逐步形成以居家养老为基础、社区养老为依托、机构养老为支撑的多元化养老格局。

　　大力发展社会事业。加强社会事业规划布局，公平均衡配置社会资源。加快教育现代化建设。切实办好学前教育，靖江、海陵、姜堰创成省级义务教育优质均衡示范区，进一步提高高中教育教学质量。加大城乡中小学教师交流力度，促进教育均衡发展。加快发展现代职业教育，推动高等教育内涵发展。建成泰州学院新校区，推进泰州职业技术学院新校区建设。深入实施市区医疗卫生资源整合，组建新口腔医院，支持兴办民营妇女儿童医院。抓好职业病防治工作。落实“单独两孩”生育政策，健全农村“三留守”人员关爱服务体系。完善体育便民设施，备战省第十八届运动会。积极发展公共交通，提升市民公交出行分担率和镇村公交运营水平。做好第三次全国经济普查工作。

　　加强和创新社会治理。深化平安泰州建设，加强社会稳定风险评估，健全矛盾排查调解体系和信访工作机制。完善社会治安防控体系，严密防范和惩治各类违法犯罪活动。推动法治泰州建设，扎实开展“六五”普法和基层民主法治实践活动；扩大法律援助覆盖面，保障法律服务需求。注重正面引导和依法管理相结合，营造良好网络环境。坚持不懈抓好安全生产，遏制重特大安全事故。健全防灾减灾救灾机制，提高应急管理能力。加强双拥共建，深入开展国防教育，做好军转安置和优抚工作。

　　5. 突出规划引领，扎实推进城镇化和城乡发展一体化。坚持以人为本，提升城镇化质量，加快形成以工促农、以城带乡、工农互惠、城乡一体的新型工农城乡关系。

　　深入推进城市建设。科学组织第四轮城市总体规划修编，切实增强规划的严肃性和引导性，真正做到尊重规划、执行规划。保护提升老城。加快凤城河风景区建设，推进稻河、高桥等街区改造复兴，加大老旧小区出新和城中村、危旧房片区改造力度。繁荣繁华新城。继续推进金融服务区、华润万象城等重点项目建设，加快完善新城生活配套设施，着力扩大文化中心的社会效益。优化完善交通。启动城市快速道路体系建设，打通梅兰路、引凤路等10条影响城市功能的“断头路”，促进区域之间的互联互通。加强城市交通管理，有效提高通行能力。建成环凤城河慢行系统7公里。抓好垃圾污水处理设施、农贸市场、停车场等配套建设，让居民生活更舒适、更方便。

　　推动城乡统筹协调发展。继续实施“城乡转型2115计划”， 突出抓好一批关键节点城镇建设。坚持以产业化带动城镇化，重视靖江、泰兴、兴化城区的规划建设，加快县域经济发展，打造一批富有现代气息、地域特色的小城市和重点镇。突出人的城镇化这一核心，推进城乡公共服务均等化。深入开展城市环境综合整治。加大农村环境整治力度，力争全面完成村庄整治任务。切实办好新一轮农村实事，认真落实扶持黄桥老区和里下河地区发展政策。

　　加快发展现代农业。大力推进国家现代农业示范区和市区北部现代农业走廊建设，不断提升农业现代化水平。创新农业经营方式，积极培育新型农业经营主体和职业农民，发展多种形式的适度规模经营。加强农产品加工集中区建设，推进主要农作物生产全程机械化，健全政策性农业保险制度，提高农业综合生产能力。实施智慧农业应用示范工程，完善农产品生产标准和质量追溯体系，让老百姓吃得放心、吃得安全。推进引江河二期、川东港兴化段、马甸水利枢纽等重点水利工程建设，实施南官河、周山河等骨干河道整治。

　　6. 深入推进文化建设，提升城市凝聚力和影响力。促进文化建设与城市建设相融合，大力发展文化产业和文化事业，进一步丰富城市内涵，彰显城市个性。

　　深化历史文化名城建设。注重传承历史文脉、保留城市记忆，落实历史文化名城保护规划。充分发掘水文化、戏曲文化、红色文化、佛教文化等特色资源，加强对名人旧居、文保建筑、古宅名园的修缮维护。加强对一批具有历史文化内涵的村落和片区的保护开发。重视非物质文化遗产保护，推进“非遗”展示馆、“老行当”历史文化街区、姜堰清明习俗文化生态保护区建设。培养 “非遗”代表传承人、传承单位，建成未成年人“非遗”传习所。

　　促进文化产业文化事业协调发展。推进文化创意产业园等园区建设，发展壮大壁纸、小提琴、工艺美术等重点产业，引进实施一批文化科技创新项目。完善国有文化资产管理体制，推动已转制文化单位建立现代企业制度。加大文化惠民力度，创建省公共文化服务体系示范区，全面实行公共文化设施免费开放。支持文艺精品创作，推出更多高质量的文化产品。办好水城水乡国际旅游节、梅兰芳艺术节等文化节庆活动。

　　提升现代文明素质。继续推进文明城市创建，做好迎接考核验收的各项工作。弘扬社会主义核心价值观，广泛开展社会公德、职业道德、家庭美德和个人品德教育，培育升华城市精神。深入开展群众性精神文明创建和“百姓”系列活动。加强青少年思想道德建设，共同营造有利于青少年健康成长的教育环境和社会氛围。加快完善社会信用体系，建立良好的诚信秩序。

　　7. 着力改善环境质量，全面加强生态文明建设。创新体制机制，形成工作合力，狠抓源头治理，不断提高生态文明建设水平。

　　严守生态安全红线。认真执行主体功能区规划，优化国土空间开发格局，确保全市生态红线面积占比不低于20%。加大环保执法力度，对污染环境、破坏生态行为严惩重罚。完善对重要生态功能区的生态补偿机制，推动地区间建立横向生态补偿制度。扎实开展生态系列创建，力争海陵、姜堰建成国家级生态区，靖江、高港通过国家级生态市（区）考核验收，泰兴进入国家级生态市考核程序，兴化通过省级生态市考核验收，全市建成省级生态市。

　　推进环境污染治理。深入实施蓝天工程，严控工业废气、机动车尾气和施工扬尘，加强秸秆禁烧和综合利用，防治大气污染。进一步完善重污染天气应急响应机制，减少雾霾天气对群众生活的影响。实施水环境质量提升工程，推进污水截管收集和集中处理，抓好城市黑臭河道整治，加强水源地保护和自来水深度处理。强化农业面源污染防治，落实最严格的耕地保护制度，确保农产品基地和农业主产区土壤安全。

　　强化生态保护修复。构建绿色生态屏障，积极打造市区北部和沿江地区绿色生态走廊，高标准推进城市绿化和农田林网建设。加强生态涵养和生物多样性保护，建好靖江马洲岛、兴化西北湖荡等生态保护区，增强生态产品生产能力。建立吸引社会资本投入生态环境保护的市场化机制，鼓励各类投资主体以多种形式参与生态环境建设。

　　8. 加强和改进政府工作，树立为民务实清廉形象。把人民群众对美好生活的向往作为政府工作的导向，不断增强政府公信力和执行力，加快建设法治政府和服务型政府。

　　推进依法行政。坚持用制度管权管事管人，把权力关进制度的笼子里。坚持科学民主依法决策，认真履行重大行政决策程序，强化决策后评价和责任追究。深化政务公开，推行政府权力清单制度，健全政府信息发布制度。自觉接受人大法律监督、工作监督和政协民主监督，主动接受新闻媒体和人民群众的监督，加强行政监督和审计监督。落实行政执法责任制，加大行政问责力度，严格规范公正文明执法。

　　切实转变作风。深入开展党的群众路线教育实践活动，全面落实改进作风各项制度规定，针对“四风”问题，突出“四查四治”，确保取得实效、取信于民。放大群众工作团效应，深化“三解三促”、“三服务”活动，尽心竭力为基层群众解决急需办、应该办、能够办的实际问题。加强学习型班子、学习型机关建设，提升履职能力和服务水平。坚持求真务实，改进文风会风，清理精减各类达标创建活动，把更多精力用在狠抓工作落实上。大兴艰苦奋斗之风，推进厉行节约反对浪费工作制度化、规范化、程序化。

　　加强廉政建设。建立健全科学有效的权力制约和协调机制，加快构建决策科学、执行坚决、监督有力的权力运行体系，完善惩治和预防腐败制度。落实党风廉政建设责任制，严肃查处腐败案件，着力解决发生在群众身边的不正之风和腐败问题。大力开展反腐倡廉教育，引导全体公务人员自觉“修剪”欲望、提升定力、抵制诱惑，从源头上预防和减少腐败现象发生，努力以良好形象赢得群众认可。

　　各位代表，全面深化改革的号角已经吹响，经济社会发展的蓝图催人奋进。让我们紧密团结在以习近平同志为总书记的党中央周围，高举中国特色社会主义伟大旗帜，坚持以邓小平理论、“三个代表”重要思想、科学发展观为指导，在省委、省政府和市委的坚强领导下，同心同德，锐意进取，扎实工作，为推进转型升级融合发展、谱写好中国梦的泰州篇章而努力奋斗！
